# Context-sensitive use of bioinformatics tools with complementary functionalities for generation of relevant hypothesis

**DOI:** 10.1186/1471-2105-15-S10-P8

**Published:** 2014-09-29

**Authors:** Vida Abedi, Mohammed Yeasin, Ramin Zand

**Affiliations:** 1 Department of Electrical and Computer Engineering, Memphis University, Memphis, TN, 38152, USA; 2 College of Arts and Sciences, Bioinformatics Program, Memphis University, Memphis, TN, 38152, USA; 3 Department of Neurology, University of Tennessee Health Science Center, Memphis, TN, 38163, USA

## Background

Bioinformatics tools can be of great help in mining and summarizing voluminous data. However, each tool has a limited array of functionalities and is targeted for niche users. Integration of bioinformatics tools with complementary functionalities, designed on different data types, can potentially enhance user experience and further knowledge discovery. We have developed a progressive approach to integrate bioinformatics tools by examining the diversity of tools that infer complementary information from the literature, high throughput genomic data and the human curated Gene Ontology classification. The goal is to build tools for inferring new and refined hypotheses for complex diseases and guide researchers towards the most fruitful directions in designing experiments and collaborating in interdisciplinary research.

## Materials and methods

**Figure 1:**
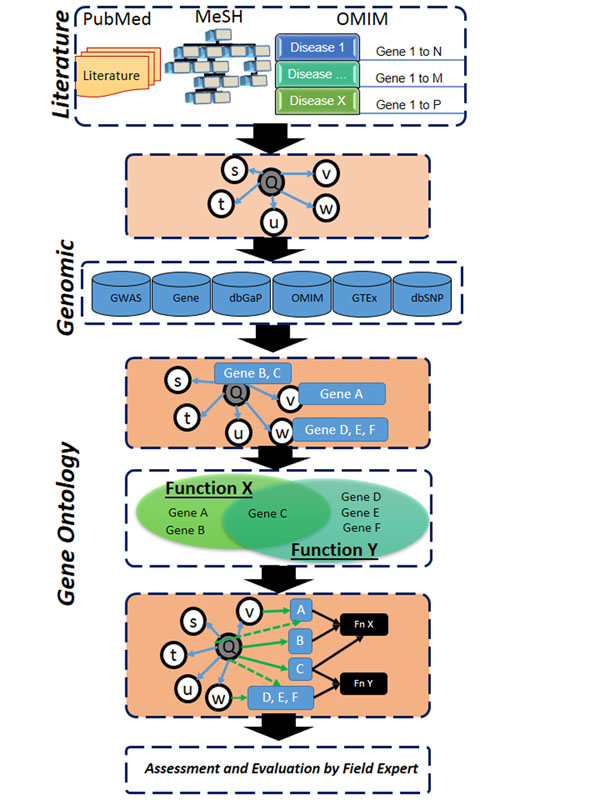
Steps of the progressive and context-specific tool integration strategy.

The proposed approach, designed to study complex diseases is summarized in Figure 1. At its root an unsupervised text analytic tool, ARIANA [1,2], is used to find the network of semantically related associations among entities – such as diseases, drugs, and pathways. At the second level, Phenotype-Genotype Integrator (PheGenI) [3] is used to extract genetic associations. At the third level, Enrichment and Functional analysis is performed using Gene Ontology (GO) [4] information through DAVID’s API functionalities [5]. The first level identifies semantically related entities to the query (indicated by “Q”). The second level utilizes the identified entities to search for associated genes from PheGenI. Extracted genes will be grouped, based on their GO, into functional groups. The functional information is added to the graph representation. Hypothesis generation is facilitated by examination of the characteristic graph. Finally, assessment and evaluation by field experts is a key step to fine-tune and enrich the new hypothesis using a direct literature search.

## Results

This pilot study provides a systemic approach to explore complex diseases using an array of bioinformatics tools. Such study could lead to tool integration. As a proof of concept, Alzheimer’s disease (AD) was explored, and an indirect association between AD and tuberculosis was identified. Matrix metalloproteinases genes and their mode of action are the origin for this association.

## Conclusions

Integration of complementary tools can help to combine functionalities and broaden services to an increasingly interdisciplinary field. The integrated system will assist the human expert and will bring hidden associations, promote data reuse, and stimulate interdisciplinary projects by connecting information across the disciplines. This may also further multi-faceted issues in knowledge discovery.
